# A Tripartite Knowledge Translation Program: Innovative Patient-Centered Approach to Clinical Research Participation for Individuals with Multiple Sclerosis

**DOI:** 10.1155/2021/5531693

**Published:** 2021-07-16

**Authors:** Aman Saini, Colleen Cochran, Audrey Zucker-Levin, Sarah J. Donkers, Pawan Kumar, Katherine B. Knox, Jessica MacPherson, Hannah Salapa, Michael C. Levin

**Affiliations:** ^1^Office of the Saskatchewan Multiple Sclerosis Clinical Research Chair, College of Medicine, University of Saskatchewan, Saskatchewan, Canada; ^2^School of Rehabilitation Science, College of Medicine, University of Saskatchewan, Saskatchewan, Canada; ^3^Department of Physical Medicine and Rehabilitation, College of Medicine, University of Saskatchewan, Saskatchewan, Canada; ^4^Saskatchewan Division-Multiple Sclerosis Society of Canada, Saskatchewan, Canada; ^5^Department of Medicine, Neurology Division, College of Medicine, University of Saskatchewan, Saskatchewan, Canada; ^6^Department of Anatomy, Physiology and Pharmacology, College of Medicine, University of Saskatchewan, Saskatchewan, Canada

## Abstract

**Background:**

Knowledge translation (KT) models that represent an individual's perspective are a sign of effective KT. Some common challenges in KT include participant engagement, organization of the team, and time demands of the participants. We implemented a unique tripartite KT program to (1) share current research, (2) inform persons living with multiple sclerosis (pwMS) about the clinical research process, and (3) invite pwMS to immediately participate in clinical research. The primary aim was to determine participants' perspectives on the value and acceptability of an experiential research program offered at a patient and family educational conference.

**Methods:**

A team of researchers identified factors that would impact the logistics of hosting an experiential research program at a conference and designed a unique tripartite KT program. The local multiple sclerosis (MS) society was engaged to select an appropriate location and invite stakeholders to the conference. A survey to determine participants' perspectives on the value and acceptability of the experiential research program was developed and analyzed.

**Results:**

65 pwMS attended the conference, and 44 (67.7%) participated in the on-site experiential research program. 72.7% of the participants completed the survey, of which 93.8% stated that they strongly agree or agree with the following statements: “Did you feel like participating in research today was a valuable experience to you?” and “Did you feel like you were contributing to MS research?” 100% of the participants agreed or strongly agreed when asked “would you like to see more research activities taking place at these kinds of events?”

**Conclusions:**

This paper describes the logistics and challenges of conducting an experiential KT program, which proved to be rewarding for pwMS. The majority of pwMS attending the conference agreed to participate in the on-site experiential research program and an overwhelming majority of participants felt the experience was valuable.

## 1. Introduction

Knowledge translation (KT) is defined by the Canadian Institutes of Health Research (CIHR) as “a dynamic and interactive process that includes the synthesis, dissemination, exchange, and ethically sound application of knowledge to improve health, provide more effective health services and products, and strengthen the health care system.” [1] An effective KT strategy should consider the micro perspective of an individual, apart from the environmental organizational view [2]. There are many KT frameworks and theories, which can be a challenge to implement [3, 4]. KT models that take into account a user's perspective are indicative of effective KT [5]. Participants who better understand the research process through experiential learning may feel differently about the value of clinical research. Experiential learning is the process of learning through experience. The association for experiential education has defined the experiential education as “a teaching philosophy that informs many methodologies, in which educators purposefully engage with learners in direct experience and focused reflection in order to increase knowledge, develop skills, clarify values, and develop people's capacity to contribute to their communities” [6]. Many factors influence how clinical research findings could be used by various stakeholders in evidence-informed decision-making [7–9]. Challenges of implementing KT from clinical research include participant or community engagement, organization of the research team, and time requirements of the participants. To address these challenges, we implemented a unique experiential tripartite KT program to (1) share current research, (2) inform persons living with multiple sclerosis (pwMS) about the clinical research process, and (3) invite interested pwMS to participate in clinical research through experiential learning at a patient and family educational conference sponsored by the Multiple Sclerosis (MS) Society of Canada. The aims were to determine participants' perspectives on the value and acceptability of an experiential research program and describe a process for developing such a program at a patient and family conference setting.

## 2. Materials and Methods

The Office of the Saskatchewan MS Clinical Research Chair was contacted by the Saskatchewan (SK) division of the Multiple Sclerosis Society of Canada (MSSC) to identify researchers to present their data in a traditional didactic setting for the “MS Connects” annual conference. The MS Connects conference is designed to bring together pwMS and caregivers, healthcare professionals, and researchers to discuss the latest advancements in MS research and symptom management. A brain-storming session identified a unique KT opportunity by using both didactic presentations and clinical research at an on-site interactive research space embedded within the conference. The goal of this project was to make the clinical research process transparent to pwMS and to actively engage the community in the research process. Considering the interest pwMS and their caregivers had shown in contacting our office about research opportunities, this would be a valuable endeavor. Further discussion regarding the logistics of hosting an event in which pwMS would participate in a research protocol at the conference identified the following components (Table 1): ethical approval, a location accessible to people with disabilities for both the didactic presentations and the data collection, preconference information to attendees regarding the opportunity for research participation during the conference, on-site provision of information to potential participants, identifying a research question that could be answered in a single visit, recruitment and training of the research team, obtaining the tools needed to perform the data collection, the layout of the conference space for conducting research (physical space for privacy, consent, participant scheduling, equipment and personal), obtaining consent, maintaining privacy, participant flow, and identification of potential bottlenecks at the conference. The study coordinator sourced all equipment needed for data collection identified by the research team and contacted the venue for equipment rental (tables/chairs/drapery, etc.). Team members were identified, and roles were assigned to spearhead individual components based on their expertise (Table 2).

The Saskatchewan Division of the MSSC helped to facilitate the plan and was responsible for securing the appropriate venue and inviting people living with MS and other stakeholders to the MS Connects conference. Upon approval by the MSSC, the clinical researchers identified a research project that could be suitable to be conducted at the conference setting and began the application for ethical approval. *We designed a project that emphasized active patient participation, which was based on the review of the existing literature and the researchers' experience. With this in mind, the team designed a research study that was relevant to individuals with MS, could be completed in a single visit, would be doable in the physical space of the venue, and would be fun for prospective participants to complete.* The approved research protocol, entitled “*A*ction *R*esearch Arm Test in *MS*” (“AR(MS)”), involved the assessment of upper extremity function, which is a significant source of disability, in pwMS. Upper extremity impairment hinders the ability of pwMS to perform activities of daily living and decreases their quality of life [10]. At the conference, we evaluated three different measures of upper extremity function: the Upper Extremity Function Scale (UEFS, a patient-reported outcome measure (PROM)), the Action Research Arm Test (ARAT), and the 9-Hole Peg Test (9-HPT), to determine which best correlates with the quality of life (QoL), as measured by the multiple sclerosis (MS) quality of life-54 (MSQoL-54) questionnaire. *The measurement of upper extremity functions and their correlation with quality of life might provide a better understanding of the disability level of patients in daily living tasks and could contribute to better planning of rehabilitation programs. We hypothesized that the multimodal ARAT may better correlate with the quality of life and the UEFS, than the 9-HPT. A peer-reviewed manuscript (based on the results of the main research study) entitled “A Descriptive Correlational Study to Evaluate Three Measures of Assessing Upper Extremity Function in Individuals with Multiple Sclerosis” was submitted to the Multiple Sclerosis International journal, which provides a detailed assessment of the data generated from this conference and our clinic.* All data were collected using REDCap, a secure web-based software platform designed to support data capture for research [ 11, 12].

**Figure 1:**
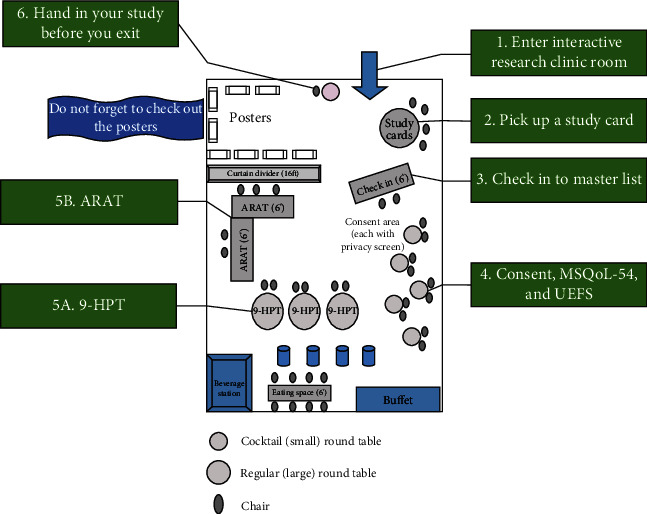
Layout of the interactive research space. Abbreviations: MSQoL-54: multiple sclerosis quality of life-54; 9-HPT: 9-Hole Peg Test; ARAT: Action Research Arm Test; UEFS: Upper Extremity Function Scale.

Ethics approval was obtained from the University of Saskatchewan's Biomedical Research Ethics Board (Protocol BIO 484). In discussions with the SK Division of the MSSC and the University of Saskatchewan's Biomedical Research Ethics Board, it was agreed that conference attendees should be given prior knowledge that the experiential research would be part of the day's events, in addition to the speaker presentations. After ethics approval, the conference was advertised on websites and social media platforms sponsored by the MSSC. To maintain confidentiality, the MSSC sent letters of invitation and information about the on-site experiential research study to pwMS and their caregivers who signed up for the conference. *The letter of invitation described the identity of the principal investigator, the purpose of the study, the study activities, the approximate time required to participate, that the data collected would be kept confidential and stored securely, and that the study was voluntary (with the requisite withdrawal from participation information). Also included in the letter was the contact information for the study coordinator so that prospective participants could contact her for detailed information about the study. The Research Ethics Office of the University of Saskatchewan reviewed and approved the invitation letter, which gave prospective participants the option to discuss their rights as a participant with an unbiased party*. Next, the floor plan of the event including the lecture space and the interactive research space was designed in collaboration with the SK division of the MSSC, lead investigators, coordinators, and venue staff (Figure 1). *It was mandatory for our team members to attend orientation and training sessions prior to the conference, which provided instruction on the proper administration of the 9-HPT and ARAT, how to properly enter data into REDCap, and manage participant flow at the event.* A research team package with a detailed description of all the activities and information was developed and distributed among the team members before the training sessions. The package also contained information on the venue, parking, timings, contact details, food and beverage, and duties assigned to the individual team members. Core team members met regularly to troubleshoot any identified barriers and test-run the protocol. Team T-shirts and name tags were designed for our team members, who arrived at the venue at a scheduled time before the start of the conference to set up their respective stations.

First, participants learned about different research topics through didactic lectures. Next, a didactic lecture reviewed the research process using the experiential learning conference project as an example study. Finally, participants were invited to partake in research at the conference, providing experiential learning, and a convenience sample of research participants was recruited from the attendees of the conference. MS researchers were selected and invited for the KT didactic presentations, which included oral lectures on stem cell therapy for MS, cannabinoids for the treatment of MS symptoms, MS research in Saskatchewan, and a novel immunotherapeutic approach for treating MS. In addition, local MS research trainees were asked to share their research via poster presentation accessible to all attendees. The research presentations were designed to provide attendees the opportunities to learn and engage with experts in the field. Following the research presentations, Michael C. Levin, M.D., the Saskatchewan Multiple Sclerosis Clinical Research Chair, gave a presentation titled “What Is Clinical Research and How to Participate in it Today.” This talk used lay language to define research, clinical research, and KT. This was followed by a detailed description of the informed consent process as well as a page-by-page review of the consent form and all of the research activities required to participate in the research protocol at the conference. He also introduced the team, which included a neurologist, a physiatrist, two physiotherapists, a nurse practitioner, nurse educators, clinic/research coordinators, a public health specialist, and research staff. *The timing of the presentation was such that prospective participants were educated about the study before being invited to participate. We arranged for the experiential research space to be open throughout the day, which gave participants plenty of time to participate. Our study team members at the consent stations also explained the consent form and answered any question individually, with dividers and appropriate spacing between stations, thus assuring patient privacy and providing ample opportunity to clarify any part of the research project*. The participants were informed that if they choose to participate in the study, there is a small chance that the upper limb mobility tasks may cause fatigue. However, all of the tasks occur while sitting, and there are medical professionals on hand if necessary. They were also informed that they may feel uncomfortable with some of the items in the questionnaires and they are welcome to answer only those they are comfortable with.

Figure 1 illustrates the layout of the interactive research space. Upon entering the interactive research area, the prospective participant was provided a study card with a unique participant identifier and a list of study activities (consent and questionnaires, 9-HPT, and ARAT). All prospective participants then checked in with a team member stationed at the check-in table. This team member was responsible for confidentially maintaining the master list with the names of participants and unique identifiers. All team members at various stations had electronic devices (computer or iPad) to collect data and were trained to log onto REDCap for entering participant's unique identifier prior to recording data. The instruments on REDCap were designed to allow confidential signing of consent mandatory prior to data fields opening for entry. After the team member confirmed the prospective participant was willing to participate in the study, they were directed to one of the five consent stations.

Each consent station was surrounded by a privacy screen and staffed by a qualified research team member. Following the electronic signature of the consent form with a stylus, participants filled out demographic information (month/year of birth, sex, year of first MS symptoms, year of MS diagnosis, MS phenotype, and current use of MS disease-modifying therapies) followed by the UEFS and the MSQoL-54. Our project was designed on REDCap in a way that the UEFS could be completed only after finishing the demographic questions and the MSQoL-54 questionnaire. Our team members assisted the participants with the tablet at the consent station and provided any other assistance required during their participation in the interactive research space. Once all the items were completed at the consent station, team members punched a hole in the study card labeled with “consent and questionnaires” as confirmation of completing this step of the study. Without having the hole punch to say that they had consented, they were unable to participate in any other study activity.

Participants were then directed to the upper extremity measurement area that included three 9-HPT and three ARAT stations. Each station was staffed by a research team member trained on administering the specific upper extremity test. *While designing the study, we took into consideration the layout of the room and the conference activities. We enabled participants to choose the activity with the shortest line, withdraw from participation at any point, and allowed participants to come and go as they pleased from the study area to attend the conference presentations. Our research team tracked participants via a study card, which was hole-punched by a team member after completion of each study activity.* Participants either self-randomized by choosing which upper extremity measure they wanted to do first or were randomized based on the availability of testing stations. For example, the first few participants were able to choose to begin with either the 9-HPT or ARAT, while subsequent participants went to the remaining unoccupied test station. Prior to testing, the participants provided the examiner with their study ID on their study card. The researcher logged into the participant's specific REDCap portfolio for data recording. Participants were instructed to return the hole-punched study card to our team members and invited to complete the participant satisfaction survey. This survey aimed at evaluating the participants' perspectives on the value and acceptability of an experiential research program *that consisted of consenting for research, filling the questionnaires on REDCap instruments (demographic, the MSQoL-54, and the UEFS), and completing the 9-HPT and ARAT*. The survey consisted of questions using a five-point Likert scale: 1 = strongly agree, 2 = agree, 3 = no opinion, 4 = disagree, and 5 = strongly disagree. Throughout the day, participants had the opportunity to review research posters, interact with our clinical research team, and ask questions related to clinical research.

## 3. Results

Sixty-five pwMS attended the conference, and 44 individuals (67.7%) participated in the on-site experiential research program. Of the 44 participants (32 females and 12 males, mean age = 49.1 ± 11.5 years, mean disease duration (in years) = 14.8 ± 13.08, 32 (72.7%) participants with relapsing-remitting MS, four with secondary progressive MS, six with primary progressive MS, and two with progressive-relapsing MS) who were consented, one participant chose not to visit 9-HPT and ARAT stations. Thirty-two participants (72.7%) completed the participant satisfaction survey (Table 3). Thirty participants (93.8%) strongly agreed or agreed with the following statements: “Did you feel like participating in research today was a valuable experience to you?” and “Did you feel like you were contributing to MS research?” Six participants (18.8%) agreed or strongly agreed to the question “Did you feel like there was too much going on and not enough time to do it all?” The majority of participants (*n* = 31; 96.9%) indicated they felt that their rights as a participant were respected and valued. Of the 31 participants who responded to the question “At any point were you uncomfortable (physically or emotionally) participating in the research activities?”, 30 participants (96.8%) disagreed or strongly disagreed with that question. All of the participants (100%) agreed or strongly agreed when asked “Would you like to see more research activities taking place at these kinds of events?”

## 4. Discussion

These data show that an experiential research opportunity was found to be acceptable and valued by pwMS attending a KT event. We also realized that the logistics of developing this type of event were feasible. We believe this approach is an effective and novel approach to KT for pwMS, and this type of program can be applied to other human diseases. There are several community participation KT frameworks, but they tend to be generic and may not take into account contextual aspects of inclusive KT [13–17]. There are only a few studies that have presented KT frameworks for the creation of evidence-based online resources for pwMS. Hill et al. started the IN-DEEP (integrating and deriving evidence, experiences, and preferences) project to produce easily accessible and meaningful evidence-based health information that could be utilized by pwMS for decision-making and self-management. Their project involved a mixed-methods approach of conducting focus groups with pwMS and their families to develop a model for presenting evidence-based information, which was later reviewed and finalized by all key stakeholders before being uploaded online and evaluated [18]. Likewise, Synnot et al. conducted a 2-phased mixed-method project for producing an evidence-based treatment information website in collaboration with pwMS. Phase 1 included review panels with pwMS and healthcare professionals to test treatment summaries (paper-based) before developing and pilot testing the website. Phase 2 included an online survey after launching the website to gather user feedback [19]. This study was limited by incomplete data ascertainment which was also observed in our study. Both of these projects described a partnership approach to developing online evidence-based information for pwMS, but these approaches lacked an experiential learning opportunity.

In this project, our team designed and successfully implemented an experiential research opportunity at a KT conference setting applicable to pwMS. This example of experiential research was useful in translating and sharing evidence-based information with key knowledge users. It could enhance comprehension and understanding of clinical research, which might lead to an increase in future engagement. It can also aid in bridging the gap between researchers and patients and potentially accelerate transformative changes in MS research that are appealing to pwMS. Community participation could also help individuals develop their knowledge, skills, and confidence to improve and gain control over the conditions that may affect their lives [20]. Importantly, pwMS and their family members are increasingly becoming active users of health information [21] and may value experiential learning opportunities. Therefore, our approach could be instrumental in improving the quality and relevance of KT for pwMS and their family members. The fact that we received positive feedback from the majority of participants suggests that we may have successfully addressed some of the common challenges in KT.

While pwMS shared that participating in experiential research combined with didactic sessions was valued, this project had a few limitations. *The use of convenience sampling might have introduced selection bias in our study. This convenience sample included individuals with MS who were active members of the MSSC or visiting the society's website and/or social media platforms and were more likely to attend the conference and volunteer for our experiential research study.* Twenty-seven percent of the participants did not complete the exit participant satisfaction survey. Incomplete data ascertainment could lead to an over- or underestimate of participant satisfaction levels. Six participants also indicated that they agreed or strongly agreed to the question “Did you feel like there was too much going on and not enough time to do it all?” These results support that some people found the study protocol challenging to complete in the time allotted. Participants were requested to complete patient demographic and quality of life survey data immediately following the consent process. This portion of the experiential research protocol took a fairly long time and involved sustained attention. Moreover, the MSQoL-54 questionnaire in itself requires considerable time to complete and could be challenging for individuals with MS suffering from fatigue. In a study conducted by Yozbatıran et al. to assess the motor function of upper extremity function and its relation with fatigue, cognitive function, and quality of life in pwMS, three patients were not able to complete a study task due to excessive fatigue and their data was excluded from the study [10]. To overcome this limitation, we could have provided flexibility to the participants for completing the questionnaires on REDCap at their convenience. Perhaps if we had scheduled a break between the consent process and the questionnaires, this may have been even more acceptable to participants. However, participants completed all aspects of the study protocol, except for one participant who did not complete all the upper limb tests, suggesting sufficient time allotted to complete this aspect of the protocol. *Another limitation is that we did not engage and consult pwMS during the design of the project. Our team had approximately three months from the initiation of the idea to the execution of the event. On reflection, if we were to host another experiential research program, we would give ourselves 6-12 months and consult individuals living with MS during the development phase.* Finally, this project did not evaluate factual knowledge gained by participants about the research process or factual knowledge gained from the didactic research topics presented. Further research would be needed to evaluate the extent of factual knowledge gained about the research process through experiential learning combined with or compared to didactic sessions alone. Considering the cost-effectiveness and accessibility of digital and remote technologies, a hybrid or a virtual model may be feasible and bring pwMS and their care providers together with researchers and health professionals from across the globe. The consent process and patient-reported outcome measures are also feasible remotely. Such endeavors could provide a more efficient and user-friendly platform for the current and future research.

## 5. Conclusions

The logistics of conducting an experiential research KT event was feasible, and pwMS found the experience rewarding. On-site experiential research program preceded by didactic sessions provided a highly acceptable platform that facilitated interactions between knowledge users and creators. The majority of pwMS attending the conference agreed to participate in the experiential research program, and an overwhelming majority of participants felt the experience was valuable and should be continued.

## Figures and Tables

**Table 1:** Logistics required for the knowledge translation program.

(i) Preconference
(a) Ethical approval
(b) Accessible location for persons with disabilities
(c) Education about how an interactive research space works
(d) Identifying a research question that can be answered in one visit
(e) Recruitment and training of the research team
(f) Identifying a unified data input tool (REDCap)
(g) Layout
(h) Documentation of participation at each research activity
(i) Advertising the conference
(ii) At the conference
(a) Education on clinical research and KT and how to participate
(b) Obtain consent
(c) Maintain privacy
(d) Flow of participants
(e) Data entry and collection
(f) Identify potential bottlenecks
(iii) Equipment required for the interactive research space; item (number)
(a) Name tags
(b) Signage
(c) Tables and chairs
(d) Curtain divides and privacy screens
(e) Easels and boards for research posters
(f) Participant study cards
(g) Printed consent forms
(h) Participant satisfaction surveys
(i) 9-HPT (3)
(j) ARAT (3)
(k) Master list
(l) iPads (5)
(m) Laptops for 9-HPT (3)
(n) Laptops for ARAT (3)
(o) Extension cords (6)
(p) Hand sanitizer (10)
(q) Sanitizing wipes (2)
(r) Hole punch (9)

**Table 2:** Number of team member(s) assigned to a specific role/research activity.

Role/activity	Number of team member(s)
Study coordinator (organize all preconference activities, lead role at conference)	1
Assistant coordinator (assist study coordinator, particularly at the conference)	1
Hand out study cards and assign research participant number	2
Obtain consent and assist with questionnaires (MSQoL-54 and UEFS)	5
Conduct 9-HPT	3
Conduct ARAT	3
Technical support	1
Obtain survey	2
Total number of research team	18

Abbreviations: MSQoL-54: multiple sclerosis quality of life-54; 9-HPT: 9-Hole Peg Test; ARAT: Action Research Arm Test; UEFS: Upper Extremity Function Scale.

**Table 3:** Results of the participant satisfaction survey.

Question(s) (*n* = total number of participants who responded to the question)	Number of participants				
	Strongly agree	Agree	No opinion	Disagree	Strongly disagree
Did you feel like participating in research today was a valuable experience to you? (*n* = 32)	25	5	1	0	1
Did you feel like you were contributing to multiple sclerosis research? (*n* = 32)	27	3	2	0	0
Would you like to see more research activities taking place at these kinds of events? (*n* = 32)	29	3	0	0	0
Did you feel like there was too much going on and not enough time to do it all? (*n* = 32)	3	3	8	7	11
Do you feel like your rights as a participant were respected and valued? (*n* = 32)	28	3	1	0	0
At any point were you uncomfortable (physically or emotionally) participating in the research activities? (*n* = 31)	0	0	1	1	29

## Data Availability

The data used to support the findings of this study are available within the article.
